# Psychometric Properties of the Italian Version of the Embodied Sense-of-Self Scale

**DOI:** 10.3390/brainsci13010034

**Published:** 2022-12-23

**Authors:** Andrea Patti, Gabriele Santarelli, Ottone Baccaredda Boy, Isotta Fascina, Arianna Ida Altomare, Andrea Ballerini, Valdo Ricca

**Affiliations:** Psychiatry Unit, Department of Health Sciences, University of Florence, 50134 Florence, Italy

**Keywords:** embodiment, psychosis, schizophrenia, Sense-of-Self, self-disorder

## Abstract

(1) Background: The Embodied Sense-of-Self Scale (ESSS) is the only validated measure for self-assessing embodiment abnormalities, which differentiate people with anomalous embodied self-representations such as schizophrenic patients from controls. The aim of the current study was to translate the ESSS from English to Italian and to examine its factor structure, reliability, and validity in the Italian context. (2) Methods: We tested the fit of the original three-factor structure (agency, ownership, and narrative identity) across a community sample (N = 269) and the reliability as well as the convergent and divergent validity of the ESSS. (3) Results: The three-factor structure of the ESSS was confirmed. However, three different factors have emerged from our analysis (self-recognition, self-consistence, and self-awareness). Higher internal consistency of the ESSS was obtained by removing six items that seemed problematic. The three ESSS scales show highly intercorrelated constructs. The measure was reliable and positively correlated with schizotypy (via the Perceptual Aberration Scale) and aberrant salience (via the Aberrant Salience Inventory), and negatively correlated with empathy (via the Italian Short Empathy Quotient scale), generalized self-efficacy (via the Generalized Self-Efficacy Scale), and social self-efficacy (via the Perceived Social Self-Efficacy Scale). (4) Conclusions: The 19-item Italian version of the ESSS is a suitable measure with which to assess embodiment abnormalities in Italian samples.

## 1. Introduction

The standard approach to one’s critical evaluation of reality is based on a three-way paradigm that comprises the concepts of space, time, and personhood. This paradigm is incorporated in a series of questions that constitutes the framework of the initial part of a psychiatric evaluation, as it represents a gateway to interpreting the cognitive faculties of an individual [1]. Given that space and time are measured quantitatively, given that they are somewhat less prone to misinterpretation and subjectivity, personhood constitutes the most variable and idiosyncratic concept.

To overcome this problem, attempts have been made to measure the “Sense of Self” via standardized, objective methods by studying the construct from a neurobiological and behavioral point of view. It is now clear that the capacity to recognize oneself is central to individual consciousness and cognition, as only the most sophisticated brains in the animal kingdom possess this feature [2].

So, the sense of self is a complex concept that should be observed from different points of view. At first, we could refer to this concept as examining the body’s representation in the brain, which is constituted by the integration of visual, tactile, and proprioceptive information. In other words, the confluence of sensory inputs from diverse sources forms a body schema and image in the mind. These composite images allow us to perceive and be aware of our body parts in space and to act and interact with the external environment. In fact, they allow us to be aware of our own bodies (as suggested by the ownership construct) and our own actions (in the form of the sense of agency). This neural mechanism is critical to building and maintaining our body representations and, inevitably, self-consciousnesses. These things considered, the sense of one’s body is also intimately related to the sense of self and could be considered the starting point to the outlining of individual psychological identity; in fact, it is the scaffold that enables us to interpret the perceptions of the inner and outer worlds, processing them into subjective feelings and representations that, in turn, dictate our behavioral patterns. 

At this point, it is interesting to underline that self-recognition requires the employment of all human senses and a multitude of mental procedures, which, when defective, can collude with mental integrity. A distorted image of the self may often be an indirect signal of an underlying psychopathology; similarly, distress in the representation of reality can be reflected in self-representation.

The human mind can be tricked into thinking that a fake hand belongs to the owner’s own body [3], and vice versa, but this holds true only if specific circumstances are met; higher systems awareness and proprioception interplay generally provide a strong enough scaffold that prevents the embodied sense of self from slipping from one’s control [4]. A scarce mentalization capacity can be explained as a failed attempt to understand oneself through integrated, intentional functions; such a deficit may be a constitutive element of the psychopathology of certain disorders and, consequently, provide a viable option to trace their trajectory and provide early intervention [5]. These considerations also hold true for the psychosis spectrum, even if the interplay between several factors is what eventually leads to the overt disease. In our framework, delusions appear to be a miscarried interpretation of one’s abnormal experiences of the embodied self, others, and/or the world. Hallucinations, on the other hand, seem to arise from the abnormal prominence of one’s bodily sensations and/or internal representations of percepts and memories [6].

In light of these considerations concerning the embodied sense of self, the possibility of employing a scale that investigates the separate dimensions of this construct, as well as the construct itself as a distinct unit, represents an objective worth pursuing. Among the existing options, it seems appropriate to select an instrument that focused on dimensions pertaining to both physical and psychological dimensions in a fluid and natural continuity. 

Asai’s Embodied Sense-of-Self Scale [7] suited all the characteristics seemingly appropriate to aim for; in the original validation study, it showed good internal reliability (the Cronbach’s α for total scale was 0.84) and validity (it showed a positive correlation with schizotypy and the sense of agency, and a negative correlation with measures of self-efficacy and self-esteem). Therefore, it constituted a scale worth translating into and validating in Italian. The scale allows for the characterization of three distinct factors, namely, ownership, agency, and narrative identity. 

Ownership and agency are embodied, non-conceptual experiences that have been distinguished as follows:

- Sense of ownership: the pre-reflective experience or sense that I am the subject of the movement (e.g., the kinaesthetic experience of movement).
- Sense of agency: the pre-reflective experience or sense that I am the author of the action (e.g., the experience that I am in control of my action) [8]

The ownership construct stems from considerations of the Rubber Hand Illusion (RHI) experiment, which, in recent years, have been expanded in new, promising, and fascinating ways. Alternatively, the sense of agency’s characterization requires the recognition of intentional patterns for its definition. Hence, when defective, it might prove potentially harmful for the operative processes of the individual, while also being strongly correlated with psychotic manifestations. For example, in schizophrenic symptoms of delusions of control or thought insertion, the sense of ownership may be retained in some form, but the sense of agency is missing. Lastly, narrative identity appears to be a higher-order, conceptually informed experience that integrates the previous first-order experiences of ownership or agency in a fluid, personalized fashion, which is best defined as the “self-labeling” of sensory information. Alterations in one’s embodied sense of self imply a global modification of the experience of the environmental and intersubjective world involving not only anomalies of object perception and meaning-bestowing but also obstacles to the intercorporeal emotional attunement between the self and other persons [9].

The ESSS has been used to discriminate schizophrenic patients, people known to have an anomalous embodied self-representation, from healthy controls. However, body–self disturbances have not only been found in schizophrenic patients but in individuals with schizotypal personality traits [10] and in psychosis-prone subjects as well [11]. Alterations of embodiment dimensions during the RHI experiment appear to also be linked with temperamental dimensions of high Novelty-Seeking (NS) and low Harm-Avoidance (HA) [12] behaviors, as measured by Cloninger’s Temperament and Character Inventory (TCI-R) [13]. This has been explained by proposing that high NS and low HA may mediate the acceptance of novel and strange bodily experiences, thus being a component in the emergence of body schema and body image dissociations.

The abundance of dimensions explored by the embodied sense-of-self construct and the relative scale, as well as their aforementioned clinical utility, inherently precipitated the explorative and validation processes outlined in the following sections.

## 2. Materials and Methods

### 2.1. Participants and Procedure

#### 2.1.1. Italian Adaptation of the Scale

Two native English speakers independently translated the original English items into Italian and resolved disagreements via discussion. The Italian items were then back-translated into English by two other researchers, who were not aware of the original scale. Afterwards, the final wording of the Italian items was determined. The translation of the scale was executed by avoiding excessively complex terms and favoring a simple syntactic formulation. No adaptations have been made for specific dialects, since, in Italy, despite the presence of numerous dialects, the Italian language is well-understood by the general population.

#### 2.1.2. Sample Size

In the original validation article, Asai relied on a sample of 718 participants to develop the 25-item scale and a sample of 106 participants to assess convergent and divergent validities. For the purpose of this work, we opted to follow Nunnally, who recommends an ideal ratio of 10 respondents per item [14]. As a result, our a priori-targeted sample size was at least 250 participants.

#### 2.1.3. Participants

Participants were recruited using convenience and snowball-sampling methods, provided they met the following inclusion criteria: they were aged between 18 and 75 years, Italian speakers, achieved a high-school degree or above level of scholarship, signed written informed consent, were Italian nationals, and resided in Italy. Exclusion criteria included illiteracy or inability to provide consent or to complete the survey online, lifetime diagnosis of a psychiatric disorder or substance use disorder, and the current use of psychopharmacological therapy. Based on the methodology adopted, a set of 45 participants was initially selected to reduce selection bias associated with the non-probabilistic sampling method. The first subjects were selected by sharing the research protocol in the University of Florence’s social spaces. Each participant was then asked to choose five individuals and to send them the questionnaire. This recruitment procedure was carried out until data were saturated.

A questionnaire comprising six different scales (see Section 2.3. Instruments), which were selected according to our starting hypotheses (see Section 2.2.1. Summary of hypotheses), was administered to each of the participants; each scale had been previously validated in the Italian language, with the contextual exception of ESSS. The questionnaire was distributed through the use of a link developed using the “Google forms” platform, which was to be filled in anonymously online from a mobile phone, tablet, or computer. The procedure was explained to respondents, and online written informed consent was collected before enrolment. The anonymity of participants was always ensured. 

In total, 309 Italian adults were recruited after providing informed consent. The following demographic and socio-economic data were collected: age, gender, marital status, education, profession, psychiatric diagnosis, and use of psychopharmacological drugs. A total of 282 participants correctly completed the survey, 15 participants did not complete the survey, 8 missed at least one response, and 4 did not complete the retest and were excluded from the study. From the 282 participants who completed the survey and the retest, another 13 were excluded (11 had a lifetime diagnosis of a psychiatric disorder or substance use disorder and 2 were using psychopharmacological drugs); thus, the final sample consisted of 269 participants, aged between 18 and 72 years old. 

All participants were retested after three months to verify the stability of the construct of embodiment over time. A long-term retest interval was chosen to avoid bias due to the short-term retest interval (e.g., participants remembering the answers, motivational factors, etc.). Data were collected from December 2021 to July 2022. The Google Forms platform was used for data collection. The study protocol was approved by the local Institution Ethics Committee (Comitato Etico Area Vasta Centro; approval code CEAVC_14709).

### 2.2. Current Study

For the purpose of this study, Asai’s Embodied Sense-of-Self Scale (ESSS) was translated into Italian, and its psychometric properties were tested on a sample of Italian individuals in order to validate its use in Italy. Currently, Asai’s Embodied Sense-of-Self Scale is the only validated measure used to self-assess embodiment abnormalities, which are usually evaluated through semi-structured interviews such as the Examination of Anomalous Self-Experiences (EASE) [15], the Abnormal Bodily Phenomena questionnaire (ABPq) [16], and the Autism Rating Scale (ARS) [17], which are tools originally designed to measure wider constructs and may incur long completion times.

#### 2.2.1. Summary of Hypotheses

**Hypothesis** **1.** The original validation article found a positive correlation between ESS and a multidimensional schizotypy scale, which has not yet been validated in Italian. For the purpose of this study, it was hypothesized that ESS could be positively correlated with the Perceptual Aberration Scale (PAS) [18], a tool that mainly focuses on bodily and perceptual experiences, which has been used to evaluate schizotypy levels both in clinical and community samples.

**Hypothesis** **2.** The author of the scale defines the body as the physical boundary between the self and the environment. Since schizophrenia involves intertwined alterations in the experience of both the lived world and lived body [19], it was hypothesized that ESS could be positively correlated with anomalous world-experiences such as those measured by the Aberrant Salience Inventory (ASI) [20], which, in turn, were found to be related to positive schizotypy both in clinical and community samples and to be an index of proneness to psychosis.

**Hypothesis** **3.** Minimal bodily selfhood has a dual function: on the one hand, it constitutes the basic sense of the self, while on the other, it shapes our perception and pre-reflective conception of others [21]. For these reasons, it was hypothesized that ESS could be negatively correlated with measures of global self-efficacy, perceived social self-efficacy, and empathy. The original validation article found a negative correlation with the self-efficacy scale (Generalized Self-Efficacy Scale, GSES) [22], which we tried to replicate in this study by using the same tool. Moreover, the original validation article found a negative correlation with a measure of empathy that has not yet been validated in Italian; thus, for the purpose of this study, we used the Italian Short Empathy Quotient scale (EQ-short) [23]. Furthermore, since social interaction is an embodied practice and the social world of persons with schizophrenia emerged as an overall crisis of embodied emotional attunement [24], it was hypothesized that ESS could be negatively correlated with the Perceived Empathic Self-Efficacy Scale (PESE) and the Perceived Social Self-Efficacy Scale (PSSE) [25].

### 2.3. Instruments

#### 2.3.1. Embodied Sense-of-Self Scale (ESSS)

Embodied Sense-of-Self Scale (ESSS) [6] is a 25-item, self-reported scale covering three factors: Ownership, Agency, and Narrative identity. The first factor includes nine items, whereas the last two include eight items each. Each item has a five-point Likert scale: (1) Strongly disagree, (2) Disagree somewhat, (3) Neither disagree nor agree, (4) Agree somewhat, and (5) Strongly agree. Therefore, the interval score for each item is 1–5, while the interval score for the three factors is 9–45 for Ownership and 9–40 for Agency and Narrative Identity. The total score ranges from 25 to 125. 

#### 2.3.2. The Perceptual Aberration Scale (PAS)

The Perceptual Aberration Scale (PAS) [18] is a 35-item, self-reported scale that investigates schizotypal traits, focusing on aberrations and distortions in perception of the body and other objects. PAS has 35 True/False items; four items are reverse-keyed and the score range is 0–35. The items were designed to explore aberrant experiences such as undefined boundaries of the body; perception of changes in spatial parameters, size, and proportions of body parts; and feeling of unreality with respect to one’s body. Since distortion of body image and unusual sensory experience were identified as schizotypy signs in several studies, higher PAS scores indicate a vulnerability for schizophrenia or psychosis. 

#### 2.3.3. The Aberrant Salience Inventory (ASI)

The ASI [20] is a 29-item self-reported scale with dichotomous answers (“Yes”/“No”) which evaluates salience attribution, which has been divided into five factors, namely increased significance, senses sharpening, impending understanding, heightened emotionality and heightened cognition. The construct itself proved to be a reliable predictor of psychosis-proneness. Scores range from 0 to 29; higher scores identify a higher psychosis proneness; moreover, score of 14 is suggested to be the cut off value to this proneness.

#### 2.3.4. The Generalized Self-Efficacy Scale (GSES)

The GSES [22] is a 10-item, self-reported scale wherein each item has a four-factor Likert scale: “exactly true (4)”, “moderately true (3)”, “hardly true (2)”, and “not at all true (1)”. The total score ranges between 10 and 40 with a higher score indicating higher self-efficacy. The GSES evaluates the way in which the subject perceives their own abilities and how they indirectly influence the subject’s specific actions and objectives. Self-efficacy can be of two types: prospective and operational. This domain encompasses both cognitions, affect, and behaviors. The GSES score also measures the ability of a subject to deal with sudden and unscheduled stressful situations that require good self-control.

#### 2.3.5. The Italian Short Empathy Quotient Scale (EQ-Short)

The EQ-short [23] is a 21-item, self-reported scale with 6 filler items and 7 reverse items. Each item has a four-point Likert scale: “true”, “fairly true”, “fairly false”, and “false”. The scale used is divided into three dimensions: Cognitive Empathy (CE), Emotional Reactivity (ER), and Social Skills (SS). The higher the total score on the EQ-short scale, the greater the ability of the subject to identify and describe the emotions of the other; vice versa, the lower the score and the greater the difficulty in identifying with others. 

#### 2.3.6. The Perceived Empathic Self-Efficacy (PESE)/Perceived Social Self-Efficacy Scale (PSSE)

The PESE/PSSE [25] is a 10-item, self-reported scale in which each item has a five-point Likert scale (1 = not well at all to 5 = very well). It is divided in two subscales: the Perceived Empathic Self-Efficacy Scale (PESE) evaluates the empathic capacity with respect to the “other”, while the Perceived Social Self-Efficacy Scale (PSE) examines the ability of the individual to build interpersonal relationships or their capacity for initiating social contact and developing new friendships. Empathy itself indirectly conditions social functioning, the aptitude for “problem solving”, and adaptation skills such that the two constructs evaluated by the two scales are interrelated. The score on these scales is associated with self-esteem and the ability to implement effective coping strategies.

### 2.4. Data Analysis

Pearson’s correlation coefficient was calculated to evaluate validity.

Reliability was assessed by estimating Cronbach’s alpha coefficients for total scale, and subscales were calculated to assess reliability.

Test–retest reliability was assessed via estimation of intraclass correlation coefficient. Intraclass correlation coefficient estimate was calculated based on a mean rating, two-way mixed effects model..

Exploratory Factor Analysis was conducted using scree test to select the number of factors in accidence with evidence in the literature showing the good reliability of this method [26]. Accordingly, the decreasing curve of the eigenvalues was represented, and the factors preceding the flattening of the curve were selected. Orthogonal rotation (VARIMAX) was performed.

Confirmatory Factor Analysis (CFA) was performed using maximum likelihood estimation. Root-Mean-Square Error of Approximation (RMSEA), Standardized Root-Mean-Square Residual (SRMR), Comparative Fit Index (CFI), and Tucker–Lewis Index (TLI) were calculated. Indices of acceptable fitness are values below 0.08 for SRMR and RMSEA, values above 0.9 for CFI, and above 0.95 for TLI [27].

Statistical analyses were performed using IBM SPSS 25.0 and AMOS 24 [28], with *p* values < 0.05 indicating statistical significance.

## 3. Results

### 3.1. Descriptive Statistics

The final sample consisted of 269 subjects, of which 156 (58%) were female and 113 (42%) were male. The mean age was 32.6 ± 11,6 years and the mean level of schooling was 18.2 ± 3.3 years. Regarding their professional statuses, 14 (5.20%) were unemployed, 65 (24.16%) were students, and 190 (70.63%) were employed. As for their sentimental statuses, 91 (33.82%) were single and 178 (66.17%) had a stable relationship. A total of 193 (71.74%) subjects did not have psychiatric familiarity and 76 (28.25%) had positive psychiatric familiarity. All the subjects resided in Italy and were native Italian speakers. Therefore, the sample excluded linguistic minorities present in Italy and non-Italian speakers (for example, the German minority of Alto Adige and the Slovenian minority of Venezia Giulia), as well as the non-native-Italian-speaking foreign population residing in Italy. The mean scores of each administered scale are shown in Table 1. The mean ASI total score was 12.49 ± 6.64, which was below the cutoff of 14 that is indicative of being prone to psychosis. However, in the total sample, 169 subjects (62.82%) had an ASI ≤ 14, while 100 subjects (37.15%) had an ASI > 14, thus indicating vulnerability to psychosis.

### 3.2. Factor Structure

Before evaluating the validity and reliability of the ESSS, a CFA was carried out to test if the original three-factor model fit the Italian Version of the scale. The CFI for the original three-factor model was 0.740, the SRMR was 0.074, the RMSEA was 0.081, and the TLI was 0.690, showing inadequate model fitness with respect to the data. Therefore, an EFA was carried out to evaluate factor structure of the Italian version of the scale; prior to this, communalities were evaluated, as shown in Table 2, and items showing communalities <0.2 were removed according to Child, 2006 [29]—this led to the removal of items no. 5, 17, 21, 23, 24, and 25.

**Figure 1:**
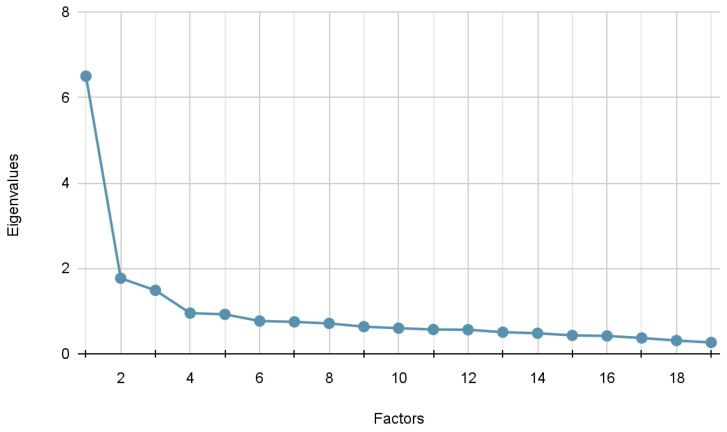
Scree test used for the EFA.

The 19-item scale was then tested with respect to factor structure. Three factors showed eigenvalues >1 (6499, 1769, and 1485) and the scree test showed that three factors preceded the flattening point of the graph, as shown in Figure 1.

The three-factor model explained 51.33% of the total variance. The factor loadings for the three factors are shown in Table 3.

The first factor, self-recognition (ESSS-R), includes eight items (1, 2, 3, 4, 6, 7, 8, and 9). This factor reflects embodied aspects of psychic, coenesthetic, and existential self-apprehension, examining the ability to recognize internal or external objects as real or belonging to the self/non-self, thus investigating experiences of depersonalization and derealization. Concerning the factors originally identified by the author, they consist of two items of agency, three items of ownership, and three items of narrative identity. Impairments in self-recognition are a common feature of various disturbing experiences in patients with schizophrenia [30], people with an at-risk mental state for psychosis (ARMS), hallucination-prone individuals, and unaffected relatives of patients with schizophrenia [31].

The second factor, self-consistence (ESSS-C), includes seven items (10, 11, 12, 13, 14, 15, and 16). This factor reflects aspects of the temporal homogeneity of one’s personality, actions, and thoughts, investigating the continuity (i.e., temporal extension through autobiographical memory) and uniformity (i.e., uniqueness known as identity, personality, and behavioral traits) of the embodied self. Concerning the factors originally identified by the author, they comprise two items of agency, three items of ownership, and two items of narrative identity. A reduced sense of personal continuity over time is a core symptom of schizophrenia [32]. In schizophrenic patients, poorer neurocognitive function and a higher degree of negative symptoms were related to less causal coherence and lower self-continuity [33]; in fact, patients with schizophrenia had significant difficulty perceiving their past, present, and future selves as unified over time compared with the controls [34].

The third factor, self-awareness (ESSS-A), includes four items (18, 19, 20, and 22). This factor reflects the sense of coenesthetic and proprioceptive connection with the body, that is, the feeling of being consciously aware of one’s own body. Concerning the factors originally identified by the author, they comprise two items of agency and two items of narrative identity.

A confirmatory factor analysis was carried out for the described three-factor model: the CFI was 0.910, the SRMR was 0.061, and the RMSEA was 0.061, showing that the model demonstrated good fitness with respect to the data. However, the TLI was 0.885; therefore, although it was higher than in the original model, is was below the cut-off for acceptable fitness.

The mean, standard deviation, and item–total correlation for the scale are reported in Table 4.

### 3.3. Reliability and Validity

Regarding reliability, the Cronbach’s alpha was 0.827 for the Self-recognition subscale, 0.825 for the Self-consistence subscale, 0.763 for the Self-awareness subscale, and 0.889 for the whole scale. A large effect size (>0.50) was detected for the correlations between ESSS total and PAS, ESSS total and ASI, and ESSS-R and PAS [35]. Correlations between ESSS-C and PAS, ESSS-C and ASI, and ESSS-A and ASI showed a medium effect size (>0.30), while the correlation between ESSS-A and PAS showed a small effect size (>0.10) [35]. Correlation analyses regarding validity are reported in Table 5.

Regarding internal validity, after a retest after 3 months, the intraclass correlation coefficient was 0.921 (F (266,266) = 12.619, *p* < 0.001) for the Self-recognition subscale, 0.997 (F (266,266) = 327.439, *p* < 0.001) for the Self-consistence subscale, 0.978 (F (266,266) = 45.090, *p* < 0.001) for the Self-awareness subscale, and 0.983 (F (266,266) = 60.230, *p* < 0.001) for the whole scale.

## 4. Discussion

This is the first study to report on the Italian version of the ESSS, which is currently the only validated measure to self-assess embodiment abnormalities both in community and clinical settings. The potential of this study lies in the fact that it encourages the pioneering employment of this scale in multiple environments, allowing for an in-depth investigation of the properties of the Italian version of the questionnaire.

Disembodiment features appear to be prominent in schizotypal individuals and psychosis-prone subjects, as confirmed by the result of the present study, which showed that ESSS has a positive correlation with PAS scores and, thus, with schizotypy, as well as to anomalous world-experiences linked to psychosis proneness such as those measured by ASI. This confirms Hypothesis 1 and 2, further confirming the central role of bodily phenomena in defining the self and highlighting the intertwinement in the experience of the lived body and lived world and the possible role of disembodiment as an index of psychotic risk. The ESSS is known to differentiate schizophrenic patients from controls, but further research is needed to investigate the relationship between disembodiment and psychosis proneness and thus the clinical utility of ESSS in intercepting subjects in ARMS.

Moreover, the ESSS showed negative correlations with the GSES, PSSE, and EQ-short, indicating that embodiment impairment impacts not only perceived self-efficacy but also the subjective engagement with the social world, thus confirming Hypothesis 3. With regard to the intersubjective dimension, the ESSS seems to affect the ability to identifying others’ emotions (EQ-short), to voice one’s own opinions to others, to work cooperatively, to share personal experiences, and to manage interpersonal conflicts (PSSE). Surprisingly, the ESSS showed a negative correlation with the EQ-short but no correlation with the PESE, which is worthy of further study. This result can be explained by differentiating the capacity of the subject to describe the emotions of the other (EQ-short), which is negatively affected by ESS, from the perceived self-efficacy in responding emotionally and compassionately to others’ distress and in being sensitive to how one’s actions affect others’ feelings (PESE), on which the ESS seems to have no effect. This may help future researchers better characterize the notion of schizophrenic “dissociality” [24], a concept that is assumed to be underlined by a disorder of embodied attunement and interaffectivity (i.e., a process wherein one’s body is tacitly affected by another’s expression in one’s bodily kinesthesia and sensation) [36]. Embodiment alterations may explain the impairments in emotional recognition usually displayed by schizophrenic patients [37] and the effect of disembodiment on the empathy quotient (EQ-short) that we have highlighted. However, during their social encounters, people with schizophrenia appear to adopt an “algorithmic conception of sociality” [24] that helps them to make sense of others’ behavior and to respond accordingly, thus possibly preserving their perceived empathic self-efficacy (PESE), while affecting their perceived self-efficacy with respect to accomplishing shared activities (PSSE).

Moreover, emotions themselves result from the body’s own feedback and the circular interaction between affective affordances in the world and the subject’s bodily resonance, which may take the form of sensations, postures, expressive movements, or movement tendencies [36]. Therefore, it is not surprising that another feature of embodiment abnormalities in schizophrenic patients is their difficulty in acknowledging that a bodily sensation may be the bodily component of an emotion, a feature which appears to be linked with the discrepancy between emotional experience, expression, and recognition known as “alexithymia” [38]; in fact, schizophrenic patients may display a reduction in emotional expression while reporting increased levels of emotional experience.

While the original embodied sense-of-self scale involved 25 items and a 3-factor structure focused on agency, ownership, and narrative identity dimensions, our study suggests that 19 items and a 3-factor structure targeting self-recognition, self-consistence, and self-awareness can provide better data fitness. This result is not meant to provide a different conceptualization of the embodied sense of self per se; rather, it highlights the difficulties of radically distinguishing between agency, ownership, and narrative identity by means of a self-report. Therefore, we agree with the author with respect to suggesting the consideration of the total score of the scale, for the Italian version as well, as an indicator of the overall levels of disembodiment.

To conclude, the present study provides support for the psychometric properties of the Italian version of the ESSS. The embodied sense-of-self scale items exhibited reasonably good internal consistency and validity, and the new three-factor model showed adequate data fitness.

## 5. Limitations

The present study must be considered in light of some limitations. The sample examined was not fully representative of the Italian population, as it presented an average age below that of the Italian population and did not present an adequate representation of ethnic minorities and non-native speakers. An additional consideration lies in the fact that even native speakers might experience difficulties in interpreting some of the questions immediately, as the explored domains are somewhat unfamiliar to the general population. Such a difficulty was also contemplated in the original study, but there is no guarantee that the range of potential misinterpretations had not widened for the proposed sample. This could lead to a reduction in the possibility of generalizing the observed results. Moreover, even if the original ESSS was developed to distinguish the sensory level of self (corresponding to agency and ownership) from identity and personality (narrative identity), in our study, it appeared difficult to radically differentiate these dimensions, which somehow overlapped and then clustered into three different factors (self-recognition, self-consistence, and self-awareness), within which agency, ownership, and narrative identity appear intertwined. This limitation may be attributed to the use of a short self-report inventory, which may flatten semantic differences and render the report incapable of offering the same amount of information as semi-structured interviews.

## 6. Conclusions

The ESSS proved to be related to aberrant salience experiences, schizotypy, and low perceptions of generalized and social self-efficacy. This is in line with the view that the concept of disembodiment reflects itself in one’s experience of the world, oneself, and others. For these reasons, it is fundamental to have a measure of disembodiment in Italy. This study should be followed by more extensive research in order to evaluate the disembodiment dimensions within the Italian schizophrenic population and between different nosographic categories.

## Figures and Tables

**Table 1:** Mean scores ± standard deviation for each administered scale.

Scale	Score
ESSS	59.42 ± 15.30
PAS	5.22 ± 4.79
ASI	12.49 ± 6.64
GSES	29.27 ± 4.47
EQ-short	45.26 ± 5.25
PESE	23.61 ± 3.03
PSSE	15.47 ± 2.69

Abbreviations. ESSS: Embodied Sense-of-Self Scale, PAS: Perceptual Aberration Scale, ASI: Aberrant Salience Inventory, GSES: General Self-Efficacy Scale, EQ-short: Empathy Quotient, PESE: Perceived Empathic Self-Efficacy, and PSSE: Perceived Social Self-Efficacy Scale.

**Table 2:** Communalities between items of the Embodied Sense-of-Self Scale (ESSS).

Item	Communality
1	0.293
2	0.328
3	0.316
4	0.362
5	0.001
6	0.356
7	0.419
8	0.228
9	0.346
10	0.552
11	0.313
12	0.449
13	0.338
14	0.239
15	0.444
16	0.363
17	0.119
18	0.245
19	0.281
20	0.221
21	0.088
22	0.299
23	0.022
24	0.193
25	0.138

**Table 3:** Factor loadings for the three-factor model.

Item	Factor		
	1	2	3
1	0.646	0.190	0.034
2	0.755	0.072	0.121
3	0.642	0.302	−0.041
4	0.579	0.318	0.079
6	0.669	0.120	0.235
7	0.506	0.473	0.094
8	0.570	0.051	0.195
9	0.627	0.194	0.175
10	0.355	0.645	0.260
11	0.046	0.728	0.155
12	0.278	0.639	0.209
13	0.205	0.456	0.353
14	0.045	0.720	0.012
15	0.342	0.603	0.153
16	0.240	0.619	0.162
18	0.095	0.215	0.685
19	0.194	0.046	0.838
20	0.037	0.146	0.822
22	0.224	0.255	0.532

**Table 4:** Mean, standard deviation, and item–total correlation for the items of the scale.

Item	Mean	SD	Item-Total Correlation
1	1.68	1.114	0.470
2	1.44	0.936	0.503
3	1.53	1.004	0.505
4	1.54	1.018	0.532
6	1.52	1.045	0.534
7	1.93	1.285	0.590
8	1.91	1.206	0.413
9	1.89	1.237	0.530
10	2.15	1.278	0.693
11	3.00	1.358	0.497
12	2.36	1.375	0.611
13	3.39	1.269	0.518
14	3.02	1.324	0.427
15	2.85	1.255	0.605
16	2.45	1.459	0.548
18	2.34	1.350	0.447
19	2.03	1.244	0.470
20	2.17	1.339	0.425
22	1.70	1.043	0.483

**Table 5:** Pearson’s correlation coefficients between the psychometric indices in the study.

	**ESSS-R**	**ESSS-C**	**ESSS-A**	**ESSS total**	**PAS**	**GSES**	**EQ-Short**	**ASI**	**PESE**	**PSSE**
**ESSS-R**	1	0.610 ***	0.376 ***	0.848 ***	0.666 ***	−0.060	−0.170 **	0.551 ***	0.175 **	−0.119
**ESSS-C**	0.610 ***	1	0.475 ***	0.889 ***	0.476 ***	−0.206 **	−0.143 *	0.469 ***	0.008	−0.153 *
**ESSS-A**	0.376 ***	0.475 ***	1	0.686 ***	0.294 ***	−0.141 *	−0.060	0.328 ***	0.006	−0.028
**ESSS total**	0.848 ***	0.889 ***	0.686 ***	1	0.609 **	−0.165 **	−0.160 **	0.561 ***	0.093	−0.133 *
**PAS**	0.666 ***	0.476 ***	0.294 ***	0.609 **	1	−0.041	−0.196 **	0.562 ***	0.153 *	−0.107
**GSES**	−0.060	−0.206 **	−0.141 *	−0.165 ***	−0.041	1	0.128 *	0.022	0.138 *	0.201 **
**EQ-short**	−0.170 **	−0.143 *	−0.060	−0.160**	−0.196 **	0.128 *	1	−0.055	0.499 ***	0.484 ***
**ASI**	0.551 ***	0.469 ***	0.328 ***	0.561 ***	0.562 ***	0.022	−0.055	1	0.130 *	−0.022
**PESE**	0.175 **	0.008	0.006	0.093	0.153 *	0.138 *	0.499 ***	0.130 *	1	0.300 ***
**PSSE**	−0.119	−0.153 *	−0.028	−0.133 *	−0.107	0.201 **	0.484 ***	−0.022 **	0.300 ***	1

Abbreviations. ESSS: Embodied Sense-of-Self Scale, ESSS-R: ESSS Self-recognition, ESSS-C: ESSS Self-consistence, ESSS-A: ESSS Self-awareness, PAS: Perceptual Aberration Scale, GSES: General Self-Efficacy Scale, EQ-short: Italian Short Empathy Quotient scale, ASI: Aberrant Salience Inventory, PESE: Perceived Empathic Self-Efficacy, and PSSE: Perceived Social Self-Efficacy Scale. Statistical significance. * *p* < 0.05, ** *p* < 0.01, and *** *p* < 0.001.

## Data Availability

All data generated or analyzed during the present study are available upon reasonable request to the corresponding author.
